# Affinity Enhancement in Discrete Multivalent MegaMolecules

**DOI:** 10.1002/cbic.70411

**Published:** 2026-06-07

**Authors:** Zhaoyi Gu, Blaise R. Kimmel, Justin A. Modica, Che‐Fan Huang, Rahul K. Salaria, Sraeyes Sridhar, Neil L. Kelleher, Milan Mrksich

**Affiliations:** ^1^ Departments of Chemistry and Biomedical Engineering Northwestern University Evanston Illinois USA; ^2^ Department of Chemical and Biomolecular Engineering The Ohio State University Columbus Ohio USA; ^3^ Departments of Chemistry Molecular Biosciences and Proteomics Center of Excellence Northwestern University Evanston Illinois USA

**Keywords:** biolayer interferometry, mass photometry, multivalency, protein engineering, protein–protein interactions

## Abstract

In this study, we quantitatively investigated the binding properties of eighteen multivalent megamolecules functionalized with nanobodies targeting epidermal growth factor receptor (EGFR) or human EGFR 2 (HER2). We synthesized a series of structurally defined multivalent megamolecules containing up to six anti‐HER2 or anti‐EGFR Nbs with monovalent affinities varying by 2800‐fold. These megamolecules exhibited improvements in apparent binding affinities from nano‐ or micromolar range to picomolar or sub‐picomolar range (31‐ to ∼118,000‐fold increase). Notably, a dendritic hexavalent megamolecule achieved sub‐picomolar binding affinity, as determined by biolayer interferometry. To assess whether the enhanced affinity of this hexamer arose from its valency or extended conformation, variants with reduced valency were synthesized and exhibited lower affinity, confirming valency as the primary factor in affinity enhancement. Our research highlights the utility of megamolecules in synthesizing precisely defined multivalent structures with controlled valency and spatial presentation, providing a versatile platform for fundamental studies and development of advanced therapeutic agents.

## Graphical abstract

We design discrete protein megamolecules with precisely controlled valency (1–6 nanobodies), which exhibit strong avidity‐driven affinity enhancement toward epidermal growth factor receptor and human epidermal growth factor receptor. We systematically investigate the roles of valency, intrinsic affinity, and spatial presentation in optimizing binding interactions, and derive design rules for multivalent protein binders.

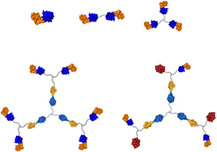

## 1 Introduction

Multivalency is an important topic in biology and drug development. The incorporation of two or more affinity domains in a protein leads to an increase in the binding affinity relative to the monovalent interaction—often referred to as avidity—and can also give enhanced specificity when the binding domains target different epitopes [1]. This principle is well understood for the binding of antibodies to cell surfaces, where bivalent binding of the two Fab domains is associated with a 10‐ to 10^4^‐fold higher affinity [2, 3, 4, 5, 6, 7, 8, 9, 10, 11]. Many other examples have demonstrated how affinity can increase further with additional copies of the affinity domains [12, 13, 14, 15, 16, 17, 18, 19], yet the study of structurally well‐defined multivalent binders is less developed. This limitation owes to the challenges in preparing structurally‐defined scaffolds that present multiple binding domains. In this work, we use the megamolecule approach to prepare a series of scaffolds that present from one to six affinity domains for either the epidermal growth factor receptor (EGFR) or human epidermal growth factor receptor 2 (HER2), and we report the relationship between dissociation constant and valency for these series.

**FIGURE 1:**
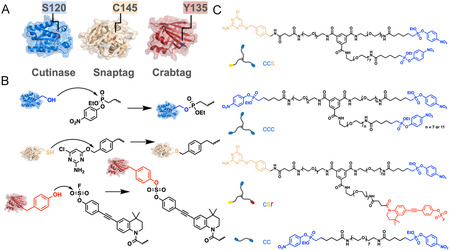
Fusion protein building blocks and linkers used to assemble megamolecule binders in this work. (A) Crystal structure of Cutinase (1CEX), SnapTag (3KZY), and CrabTag (7RY5) indicating the positions of their active sites. (B) Structures of the covalent inhibitors for each protein, showing the products that result from the reactions. (C) Structures of the linkers used to assemble the multivalent binding megamolecules used in this work. From top to bottom: ccs, ccc, csr, cc, and cs. c = Cutinase inhibitor, s = SnapTag inhibitor, and r = CrabTag inhibitor.

We have developed the megamolecule platform to prepare protein‐based scaffolds that can be functionalized with Fab and other affinity domains, and in this sense serve as antibody mimics. The scaffolds are assembled from fusion proteins and linkers, where reactions between an enzyme within the fusion and a covalent inhibitor on the linker gives a defined covalent conjugation. We have developed, and borrowed, several such reactions, including those between Cutinase and a *p*‐nitrophenyl phosphonate [20, 21], SnapTag and a chloro‐pyrimidine [22], and CrabTag and a synthetic retinoid having a fluorosulfonate group [23] (Figure 1A,B). In each case, a binding step brings the reaction partners together, even when they are present in mixtures and at low concentration, and further, the reactivity of the covalent inhibitor is increased when bound in the active site. These covalent linkages produce chemically homogeneous and stable protein constructs that resist hydrolysis and degradation in buffer [21, 23] and under physiological conditions [24, 25]. Compared to other assembly approaches that rely on noncovalent hybridization [17] and often require chemical modification of protein surfaces for attachment [26], the megamolecule platform offers distinct advantages as it forms covalently linked, all‐protein assemblies without altering the functional domains, yielding intrinsically stable and biocompatible multivalent constructs. We have used this approach to assemble very large, but perfectly defined, structures and have reported examples of antibody mimics for therapeutic applications [21, 24, 25, 27, 28, 29].

In this article, we describe the use of the megamolecule platform to prepare a series of multivalent binders that present one, two, three or six nanobody affinity domains for either EGFR or HER2. We characterized the kinetic constants for association and dissociation of these binders for their immobilized partners, and we quantitated the enhancement in affinity with increasing valency and found that the novel hexamer displayed exceptional binding capability due to its hexameric valency. This study provides an early example of well‐defined multivalent binders for understanding the relationship between affinity and valency.

## 2 Results and Discussion

### 2.1 Design and Synthesis of Multivalent Megamolecules

**FIGURE 2:**
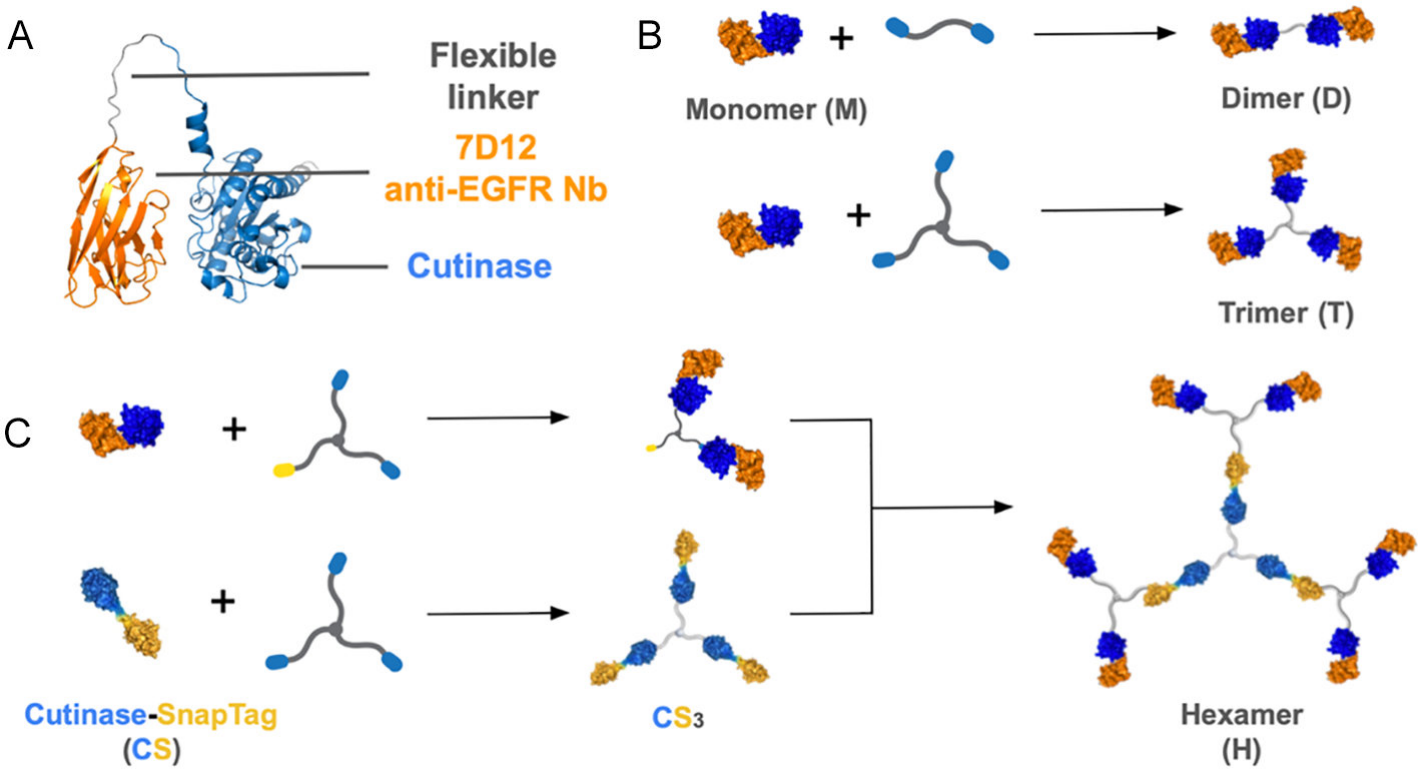
Schematic for the assembly reactions used to prepare the M, D, T, and H binding proteins. The structures of the linkers are shown in Figure 1C. (A) Model of the 7D12‐Cutinase fusion protein connected by a flexible linker. (B) Synthesis of dimer (D) and trimer (T). (C) Synthesis of hexamer (H) by assembling a tri(Cutinase‐SnapTag) (CS_3_) core with three bivalent 7D12 fragments.

We first prepared a series of megamolecules that present one, two, three or six copies of the 7D12 nanobody that binds to EGFR [30]. We first expressed a fusion protein having one 7D12 nanobody and a C‐terminal Cutinase. The nanobody and enzyme were connected through a flexible, unstructured XTEN linker, which has been demonstrated in prior studies to preserve the activities of fusion proteins [31]. This fusion protein serves as the monovalent binder (labeled as 7D12 in subsequent contexts) and is also used as a building block to assemble the multivalent molecules (Figure 2A). Toward this end, we allowed an approximately stoichiometric amount (1.1‐fold excess per arm) of this 7D12 monomer (M) to react with either a two armed (cc) or a three‐armed linker (ccc) where each arm is terminated with the phosphonate group for reaction with the Cutinase domain (Figures 1C and 2B). These reactions resulted in the divalent (D) and trivalent (T) binders, respectively, and were purified by size exclusion chromatography (SEC). Finally, we prepared the hexavalent binder (H), by first assembling a tri(Cutinase‐SnapTag) (CS_3_) core by assembling three Cutinase‐SnapTag (CS) fusion proteins on the three‐armed linker used above. We then elaborated this intermediate by attaching a fragment having two copies of the 7D12 nanobody, where the latter molecule was prepared by attaching two 7D12 domains with a tri‐heterofunctional linker (ccs) having two Cutinase and one SnapTag covalent inhibitors (Figure 2C). These synthetic linker backbones are based on polyethylene glycol (PEG), whose length and flexibility have empirically shown to be sufficient to maintain the function of binding domains [24, 25, 29].

The molecular weights of the four molecules were calculated to be approximately 38, 76, 115, and 367 kDa, respectively, and were verified by mass spectrometry and denaturing gel electrophoresis (Table S1 and Figure S2, S7). The hydrodynamic volumes for the molecules were measured by dynamic light scattering (DLS), which showed sizes of 6, 7, 10, and 17 nm for the M, D, T, and H binders, respectively (Table S1). A negative‐stain electron microscopy image of H revealed a diameter of 16 ± 1.7 nm, consistent with the size determined by DLS (Figure S3 and S12).

### 2.2 Enhanced Affinity through Multivalency

**FIGURE 3:**
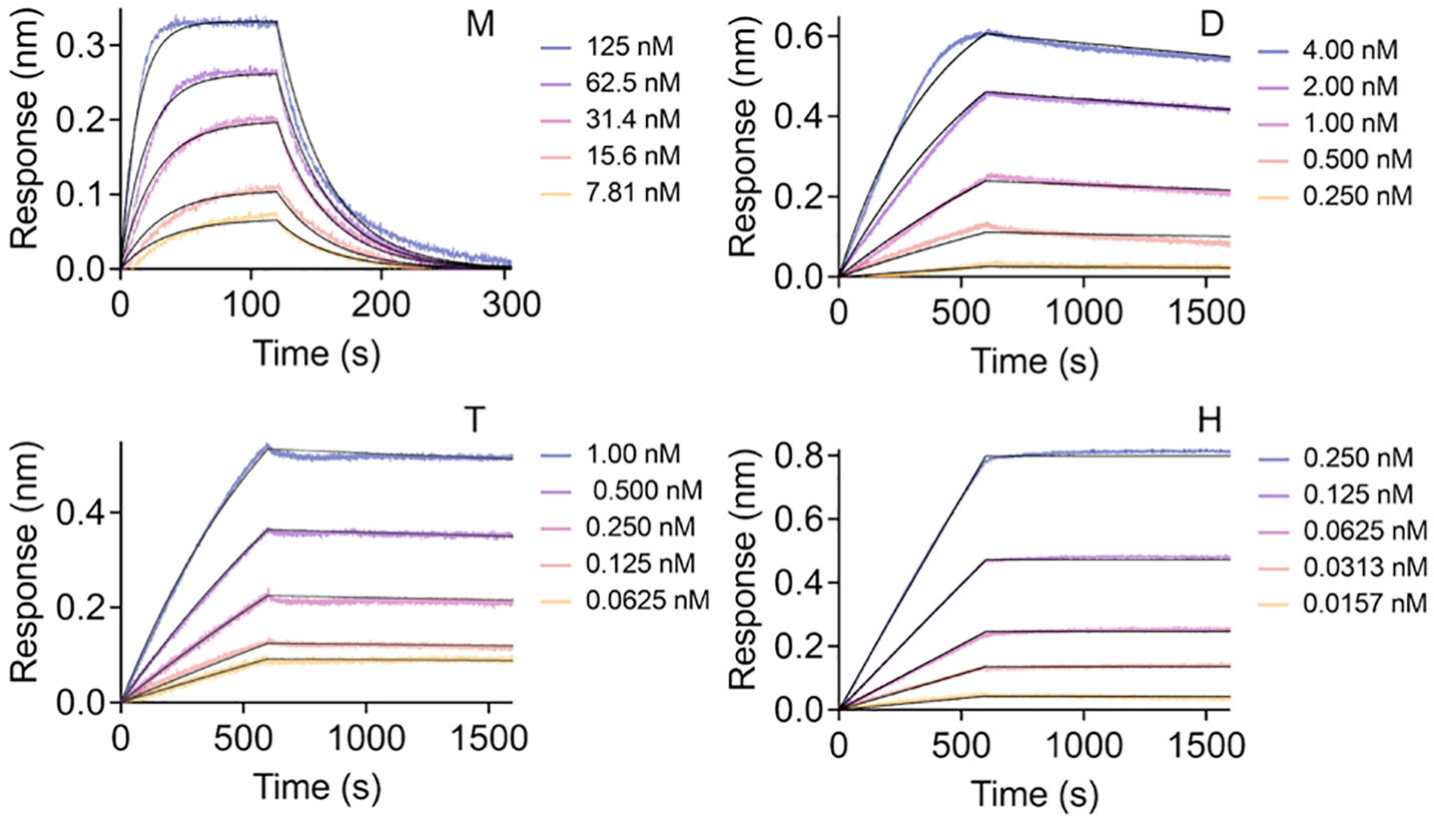
BLI sensorgram traces for association and dissociation of the M, D, T, and H megamolecules with immobilized EGFR. The timescale for the experiments were adjusted for each molecule, and analysis of these data provided the rate constants shows in Table 1.

We next measured the binding affinities for the four megamolecules using biolayer interferometry (BLI) (Figure 3). BLI is a label‐free technique that measures changes in the interference pattern of light reflected from a biosensor surface as biomolecules bind to or dissociate from it, and provides kinetic and equilibrium constants that characterize the interaction of these multivalent molecules with their immobilized ligands. We observed that the association rate increased from M to T binders, but decreased for the H binder (Table 1). The dissociation rate decreased consistently with increasing valency from M through H binders. The hexavalent binder showed negligible dissociation after 1000 s, and an off‐rate that was too slow to be measured. As a result, the K_D_ value was estimated to below 1 pM as previously reported ultra‐high‐affinity binders which also concluded a K_D_ < 1 pM due to detection limit [32, 33, 34, 35].

**TABLE 1:** Binding affinity, enhancement parameter (β), and cooperativity parameter (α) of multivalent megamolecules functionalized with anti‐EGFR Nb 7D12 to the EGFR extracellular domain (ECD), determined by BLI.

Construct	K_D_, nM	k_a_ (×10^5^ M^−1^s^−1^)	k_d_ (×10^−4^ s^−1^)	1/β	α
M	65.9	3.94	259	1	N/A
D	0.164	6.08	0.994	402	0.681
T	0.0317	12.8	0.405	2079	0.487
H	<0.001	1.11	<0.001	N/A	N/A

For each of the multivalent binders, we calculated the enhancement factor (1/β), which quantifies the improvement in binding affinity relative to the monomer (Equation (1)), and the cooperativity parameter (α), which describes how the binding of one site affects the binding of subsequent sites in a multivalent molecule (Equation (2)), as previously described by Mammen and Whitesides [36].

(1) $$K_{D,N}={\mathrm{\beta K}}_{D,1}$$

(2) $$K_{D,N}={K_{D,1}}^{\mathrm{\alpha N}}$$

In this equation, K_D, 1_ represents the binding affinity of the monovalent binder (M), while K_D, N_ denotes the binding affinity of the multivalent molecule, where N is the valency. The binding affinities and enhancement factors ‐ defined as the fold improvement in apparent affinity relative to M ‐ are summarized in Table 1. The K_D_ of D is 402‐fold (K_D_ = 164 pM), and T is 2079‐fold (K_D_ = 31.7 pM), higher than M (K_D_ = 65.9 nM). Notably, the fold enhancement (1/β) of apparent K_D_ of H (K_D_ < 1 pM), showed a more than 65,000‐fold increase. The enhancement was primarily due to a reduction in the dissociation rate constant, indicating that the multivalent interaction stabilizes the binding complex and prevents dissociation. Multiple simultaneous binding events can occur between the multivalent binder and its target, creating a cooperative stabilization of the complex. Even if one binding arm transiently dissociates, the remaining interactions maintain the complex in close proximity, thereby facilitating rapid re‐binding and effectively reducing the overall dissociation rate. This finding aligns with previous studies on multivalent systems, where increased valency typically enhances binding by increasing the lifetime of the complex, and not the rate for its formation [12, 13, 15, 16, 37]. The cooperativity parameter (α) for all constructs was less than 1, indicating negative cooperativity likely due to steric hindrance or non‐optimal conformation of the linker as observed in other multivalent systems with high affinity binders [13, 15, 36, 38]. As is typical for most multivalent interactions, including antibody binding to cells or solid supports, negative cooperativity does not preclude a strong net binding effect. Instead, multivalency‐enhanced avidity still dominates due to the additive contributions of multiple interactions, resulting in a substantial increase in overall affinity, as exemplified by H.

### 2.3 Impact of Valency and Spatial Presentation on Apparent Affinity of Multivalent Megamolecules

The hexamer format demonstrated superior enhancement in apparent K_D_, prompting us to investigate whether this improvement was dependent on the overall dimension of the megamolecule, as molecules with insufficient dimension may not be able to engage multiple binding partners due to steric interactions. Given that the size of EGFR extracellular domain (ECD) (72 kDa) is comparable to that of T (115 kDa), we hypothesized that the extended conformation of H (368 kDa) might reduce steric constraints to engage more antigens.

**FIGURE 4:**
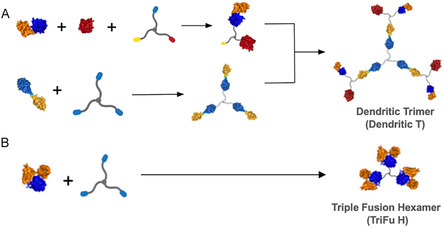
Synthesis scheme of dendritic trimer (dendritic T) and triple fusion hexamer (TriFu H). The structures of the linkers are shown in Figure 1C. (A) Synthesis of dendritic T. The CS_3_ core was reacted with three copies of a fragment comprising one 7D12 nanobody and one inactive CrabTag domain. This fragment was constructed by linking the two domains via a csr linker containing one Cutinase, one SnapTag, and one CrabTag covalent inhibitor. (B) Synthesis of TriFu H.

To test this hypothesis, we synthesized two control binders: a dendritic trimer (dendritic T) and a triple fusion hexamer (TriFu H). The dendritic T retains the branched architecture of H but replaces half of the binding domains with CrabTag domains (Figure 4A). It was assembled using a similar approach as H, except that its arms were synthesized by linking a single 7D12 domain and a CrabTag inactive domain with a csr linker containing a Cutinase, a SnapTag, and a CrabTag covalent inhibitor (Figures 4A and 1C). TriFu H was assembled by connecting three copies of triple‐fusion proteins, each containing two 7D12 domains and one Cutinase domain, with a triCutinase (ccc) linker (Figures 4B and 1C). All constructs were assembled using a similar modular approach and characterized by SDS‐PAGE, mass photometry (MP), and size exclusion chromatography (SEC) (Figure S5 and S11).

BLI measurements revealed that dendritic T exhibited a weaker binding affinity (K_D_ = 81.9 pM) compared to H (K_D_ < 1 pM) (Table 2 and Figure S5E), consistent with its lower valency. The TriFu H displayed an intermediate apparent affinity (K_D_ = 8.4 pM), stronger than that of dendritic T (K_D_ = 81.9 pM) but still weaker than the fully dendritic hexamer (H) (K_D_ < 1 pM). This trend suggests that while increasing valency enhances apparent affinity, spatial arrangement and flexibility of binding domains also play critical roles. The observed pattern aligns with the radial binding model proposed by Kitov and Bundle [39], where a radial arrangement with long, flexible linkers maximizes accessibility and the number of productive binding configurations, thereby maximizing the avidity effect.

**TABLE 2:** Binding affinity, enhancement parameter (β), and cooperativity parameter (α) of the dendritic trimer (dendritic T) and triple‐fusion hexamer (TriFu H) of 7D12.

Construct	K_D_, nM	k_a_ (×10^5^ M^−1^s^−1^)	k_d_ (×10^−4^ s^−1^)	1/β	α
Dendritic T	0.0819	1.79	0.146	805	0.468
T	0.0317	12.8	0.405	2078	0.487
TriFu H	0.008	32.4	0.252	8238	0.258
H	<0.001	1.11	<0.001	N/A	N/A

*Note:* Values for T and H are repeated from Table 1 for direct comparison.

A closer examination of the kinetic parameters revealed that the association rate constant of dendritic T (k_a_ = 1.79 × 10^5^ M^−1^s^−1^) was more similar to that of H (k_a_ = 1.11 × 10^5^ M^−1^s^−1^), whereas its dissociation rate constant (k_d_ = 0.146 × 10^−4^ s^−1^) was closer to that of T (k_d_ = 0.405 × 10^−4^ s^−1^). The difference in these rate constants suggests that while the scaffold for the H binder has a slower binding to EGFR, the overall stability of the binder–receptor interaction is primarily governed by the number of binding domains. This further supports the conclusion that valency is the key driver of apparent affinity enhancement observed for the 7D12 H binder.

**FIGURE 5:**
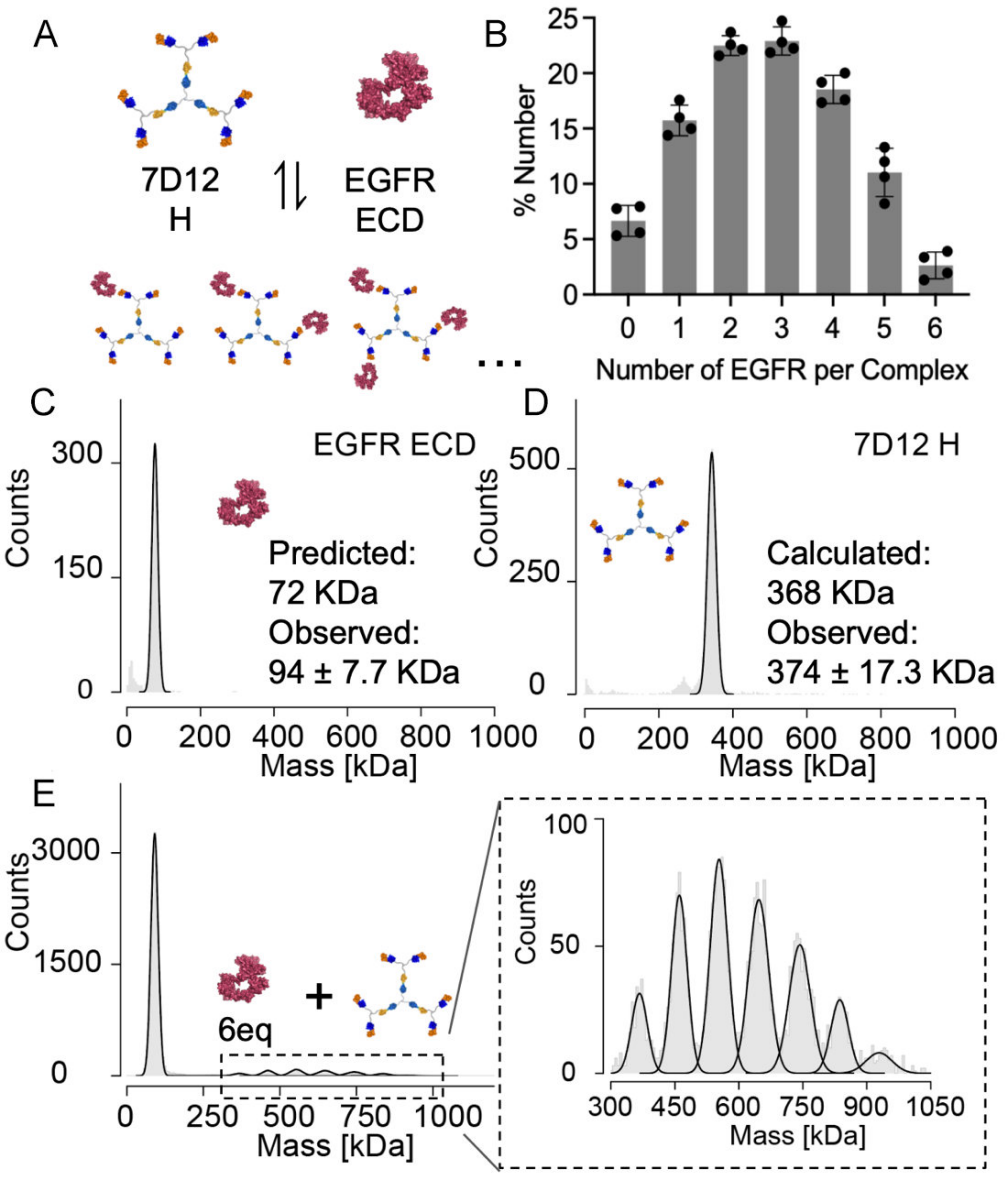
Mass photometry (MP) analysis of the 7D12 hexamer (H) in complex with EGFR. (A) Schematic representation of H binding to EGFR ECD. (B) Percentage of free H and H‐EGFR complexes determined by MP (error bars represent standard deviation). (C) Molecular weight distribution of the EGFR ECD measured by MP. The observed molecular mass is higher than the theoretical value (71.9 kDa) due to additional protein tags and abundant glycosylation [40]. (D) Molecular weight distribution of 7D12 H determined by MP. (E) MP analysis of H‐EGFR complexes when H was treated with a 6‐fold excess of EGFR ECD and zoomed‐in view of the 300–1050 kDa range.

To confirm whether H can simultaneously engage six EGFR molecules, we used MP to analyze the stoichiometry of the complexes present at equilibrium for H binding EGFR. Incubation of H with 6 equivalents of EGFR ECD showed a distribution of complexes containing up to 6 bound EGFR molecules (Figure 5), confirming the ability of H to engage up to six antigens simultaneously and establishing that steric effects in the maximally bound complex are minimal. Taken together, our findings demonstrate that valency is the primary factor driving the enhancement of apparent affinity observed for H.

### 2.4 Impact of Monovalent Intrinsic Affinity on Apparent Affinity of Multivalent Megamolecules

We next asked whether the gains in affinity with higher valency depended on the affinity of the monomeric binder—that is, whether the gain in affinity depends on the dissociation constant for the monomer? We asked this question with multivalent binders for the HER2 antigen because previous studies have reported nanobodies (Nbs) having a wide range of affinities for this protein, spanning from low nanomolar to micromolar levels [41, 42].

We generated Cutinase fusions (which again also serve as the monomeric binder, M) for three anti‐HER2 Nbs (5F7, C8, and F7) covering a wide range of monovalent intrinsic affinities (and having K_D_ values of 1.23 nM, 118 nM, and 3.37 µM, respectively) and we prepared the corresponding D, T, and H binders for each of the three nanobody affinity reagents (Table S2–S3 and Figure S6). Consistent with the trend observed in the EGFR system, the apparent affinity of the HER2 binders improved most significantly as valency increased from monovalent to divalent, with diminishing returns as the valency further increased (Table 3 and Figure S4). At the highest valency of six, the F7 H binder achieved a approximately 5450‐fold enhancement, while 5F7 and C8 reached affinity values below the detection limit (K_D_ < 1 pM) and therefore could not be quantitated. The affinity values of these H binders are consistent with predictions based on inverse power law fitted to K_D_ values of of M, D, and T (Table S4 and Figure S9). Though the fit is empirical and limited by the small dataset, the observed trend aligns with findings from Wagner et al. [13]. This plateauing effect at high valency has been also observed in other multivalent systems [15, 43, 44, 45].

**TABLE 3:** Binding affinity and enhancement parameter (β) of multivalent megamolecules functionalized with anti‐HER2 Nbs to the HER2 ECD, determined by BLI.

Nb	Construct	K_D_, nM	k_a_ (×10^5^ M^−1^s^−1^)	k_d_ (×10^−4^ s^−1^)	1/β	α
5F7	M	1.23	6.52	8.05	1	N/A
	D	0.0389	13.4	0.521	31	0.584
	T	0.0251	18.1	0.455	49	0.397
	H	<0.001	0.747	<0.001	N/A	N/A
C8	M	118	5.16	610	1	N/A
	D	0.316	19.8	6.25	373	0.686
	T	0.0692	11.6	0.804	1705	0.489
	H	<0.001	14.6	<0.001	N/A	N/A
F7	M	3370	1.38	4640	1	N/A
	D	12.5	0.944	11.8	270	0.722
	T	2.57	0.938	2.41	1311	0.523
	H	0.618	2.93	1.81	5453	0.280

We also prepared the dendritic T binder using C8, which has a similar K_D_ value to that of 7D12 (118 nM and 65.9 nM, respectively). Consistent with the EGFR binders described above, the affinity of the C8 dendritic T (K_D_ = 152 pM) was weaker than that of its H counterpart (Table S5 and Figure S4), supporting the interpretation that valency rather than spatial presentation of the binding domains is the primary factor giving affinity enhancement in the H binder.

We then analyzed how affinity enhancement correlated with intrinsic monovalent affinity. The Nbs with intermediate and weak affinities (C8 and F7) exhibited the greatest enhancement in their apparent affinity (373‐ and 1705‐fold for C8 D and T; 270‐ and 1311‐fold for F7 D and T). These two Nbs exhibited rapid dissociation in their monovalent forms, and thus benefited the most from multivalency. In contrast, the 5F7 nanobody, which has a dissociation rate constant two to three orders of magnitude lower than those of C8 and F7, showed a modest 31‐ and 49‐fold enhancement for D and T binders, respectively. These findings align with previous studies, which have shown that the reduction in the dissociation constant for bivalent interactions was inversely proportional to the strength of monovalent interaction [46]. A similar trend has been observed in systems involving small‐molecule‐modified nanoparticles, where ligands with weaker intrinsic affinity showed greater avidity effects [47]. Other studies, in which intrinsic affinities were not directly compared, have also reported affinity enhancements in the range of several hundred‐ to thousand‐fold for binders with weak intrinsic affinity (hundreds of nanomolar to micromolar range) [12, 48, 49, 50].

Taken together, our results demonstrate that the affinity enhancement observed in the HER2 system closely parallels that of the EGFR system. Valency is the primary factor driving affinity enhancement, with weak and intermediate binders benefiting most from avidity effects. Notably, the H binders showed remarkable performance, with an over 100,000‐fold affinity enhancement for C8, demonstrating the power of multivalency in overcoming suboptimal binding conditions. As we observed diminishing returns across the anti‐EGFR and anti‐HER2 binders, we anticipate only modest additional gains in apparent affinity for even higher valency molecules. The H binder therefore provides an optimal balance of affinity and synthetic feasibility.

Understanding the design rules of multivalent binders provides valuable insights for understanding biomolecular interactions and for designing new therapeutics. Future studies could benefit from fundamental simulations to explore the antigen binding dynamics and optimize linker composition to maximize binding efficiency while minimizing steric hindrance [28, 51, 52]. This work is also significant because it adds to previous descriptions of hexavalent formats [53, 54, 55, 56], by using structurally defined multivalent binders, with precise control over spatial configuration and valency. The megamolecule approach can also be extended to the evaluation of multispecific binders by using multiple orthogonal megamolecule chemistries. The hexamer formats can be adapted to various applications, including biologics with enhanced viral neutralization ability [16], multispecific or multiparatopic molecules with improved specificity and resistance to mutational escape [57, 58], and multivalent constructs made of low affinity binders to target disease‐related cells with better specificity [59, 60, 61, 62].

## 3 Conclusion

We have developed discrete multivalent megamolecules with precisely organized structures. Our results demonstrated that control over valency and spatial presentation can enhance binding affinities by over 100,000‐fold enhancement. It is important to note that optimal valency and conformation are system dependent and may differ in more complex cellular environments, where factors such as receptor density, membrane organization, and location interactions can influence binding behavior. Although similar valency‐dependent affinity enhancements have been reported in other systems [24, 63], the optimal valency and spatial configuration must be empirically determined for each specific molecular and cellular context. Collectively, these results establish a versatile and tunable framework for engineering multivalent interactions and provide a quantitative foundation for future cellular studies. This work underscores the importance of valency and spatial presentation in optimizing binding interactions and suggests promising avenues for future research and applications in drug development.

## Funding

This work was supported by the Defense Threat Reduction Agency (HDTRA1‐21‐1‐0038); National Institute of General Medical Sciences (GM108569); National Science Foundation (ECCS‐2025633, DMR‐2308691); National Institutes of Health (S10OD026871).

## Conflicts of Interest

The authors declare no conflicts of interest.

## Supporting information

The authors have cited additional references within the Supporting Information [64].

## Data Availability

The data that support the findings of this study are available from the corresponding author upon reasonable request.
