# Supplementary material for: Affinity Enhancement in Discrete Multivalent MegaMolecules
Source: Chembiochem. 2026 Jun 7;27(11):e70411. doi: 10.1002/cbic.70411 (PMC13243943; doi:10.1002/cbic.70411)
Supplement: Supplementary file 1 — The authors have cited additional references within the Supporting Information [64]. [file CBIC-27-e70411-s001.pdf]

Supporting Information for

Affinity Enhancement in Discrete Multivalent MegaMolecules

Zhaoyi Gu, [a] Blaise R. Kimmel,[c] Justin A. Modica,[a] Che-Fan Huang, [b] Rahul K. Salaria, [a] Sraeyes Sridhar, [a] Neil L. Kelleher, [b] Milan Mrksich* [a]

[a] Z. Gu, J. A. Modica, R. K. Salaria, S. Sridhar, M. Mrksich
Departments of Chemistry and Biomedical Engineering
Northwestern University
2145 Sheridan Rd, Evanston, Illinois 60208, United States
E-mail: milan.mrksich@northwestern.edu

[b] C. -F. Huang, N. L. Kelleher
Departments of Chemistry, Molecular Biosciences and Proteomics Center of Excellence
Northwestern University
2145 Sheridan Rd, Evanston, Illinois 60208, United States

[c] current address for B. R. Kimmel
Department of Chemical and Biomolecular Engineering
The Ohio State University
151 W. Woodruff Avenue, Columbus, OH 43210

Table of Contents

Additional Tables pg. S3
Additional Figures pg. S5
Sequences of MegaMolecule Proteins pg. S10
Additional Experimental Details pg. S11

TABLES

Table S1. Summary of size and molecular weight of 7D12 M, D, T, and H binders determined by dynamic light scattering and mass spectrometry.

Construct	Z-Average diameter (nm)	Calculated MW (Da)	MS MW (Da)
M	5.84	37762.87	37758 [a]
D	7.11	76270.10	76259 [a]
T	9.51	115114.55	115098 [a]
H	16.89	367541.94	367710 [b]

[a] Molecular weight of monomer, dimer, and trimer were determined by electrospray ionization mass spectrometry (ESI).

[b] Molecular weight of G1 hexamer was determined by native mass spectrometry.

Table S2. Molecular weight of M, D, T binders, and 7D12 TriFu determined by ESI.

	M		D		T	
**Nb-cut**	**Calc'd MW (Da)**	**Observed (Da)**	**Calc'd MW (Da)**	**Observed (Da)**	**Calc'd MW (Da)**	**Observed (Da)**
5F7	37027.32	37024	74799.62	74791	112907.9	112897
C8	37086.34	37081	74917.66	74907	113084.96	113072
F7	37380.46	37375	75505.9	75495	113967.32	113952
7D12	37762.87	37758	76270.72	76260	115114.55	115098
7D12 TriFu	52256.64	52250				

Table S3. Molecular weight of H, T, dendritic T, and TriFu H binders determined by mass photometry (MP).

Construct	Nb-Cut	Calculated MW (Da)	Observed (kDa)
H	5F7	363128.64	360 ± 15.8
H	C8	363482.76	362 ± 14.9
H	F7	365247.48	370 ± 13.6
H	7D12	367541.94	367 ± 11.5
T	7D12	115114.55	113 ± 10.5
Dendritic T	7D12	303385.40	301 ± 26.0
TriFu H	7D12	158597.86	150 ± 14.6

Table S4. Binding affinity (K_D) values measured by biolayer interferometry (BLI) and corresponding fits using an inverse power law model. The data was fitted using the equation $y = ax^b$, where x represents binder valency. For H binders, the valency (x) was set to 6.

Construct	Measured K_D (pM)	Fitted K_D (pM)	a	b
5F7 H	< 1	1.3	0.9603	-3.690
C8 H	< 1	0.33	87.83	-6.964
F7 H	618	16	2606	-6.699
7D12 H	< 1	0.14	49.62	-7.137

Table S5. Binding affinity, enhancement parameter (β), and cooperativity parameter (α) of the dendritic T of C8 determined by BLI.

Construct	K_D (nM)	k_a ($\times 10^5$ $M^{-1}s^{-1}$)	k_d ($\times 10^{-4}$ s^{-1})	1/β	α
C8 dendritic T	0.1524	11.53	1.756	774	0.472

FIGURES

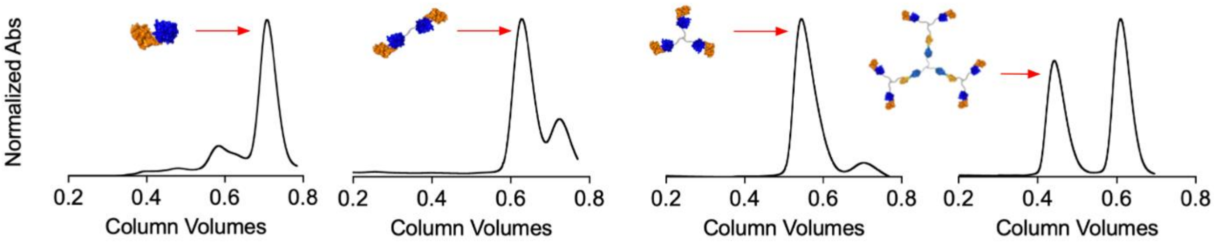

Figure S1. SEC purification traces of 7D12 M, D, T, and H.

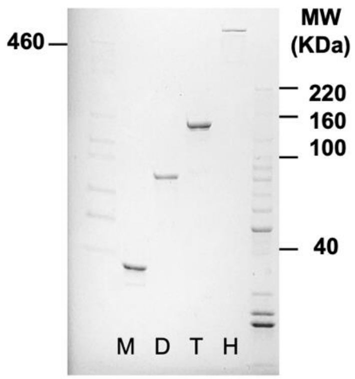

Figure S2. SDS-PAGE of 7D12 M, D, T, and H.

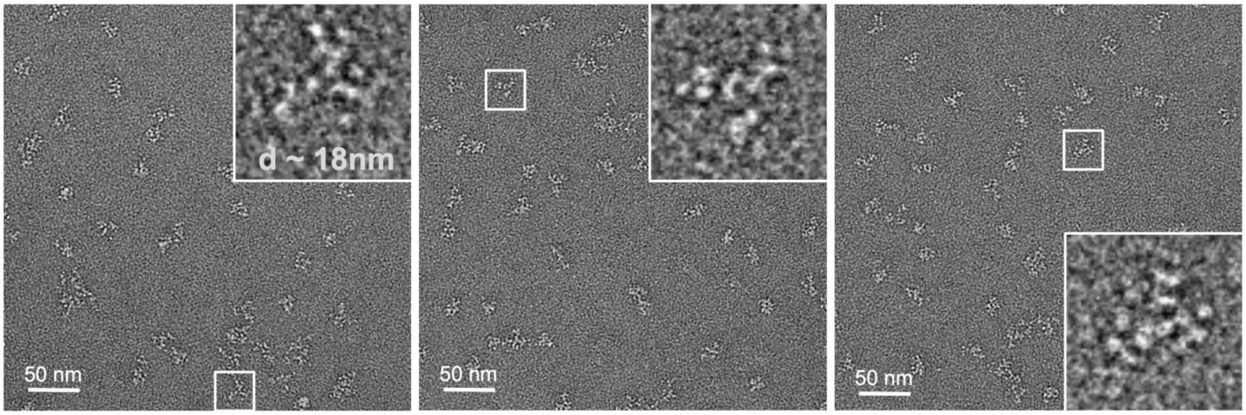

Figure S3. Representative negative-stain TEM image of 7D12 H. The enlarged particles shows molecules with relatively extended conformations, which are chosen to better visualize the fusion protein domains within individual molecules. A comprehensive particle size analysis is presented in Figure S12.

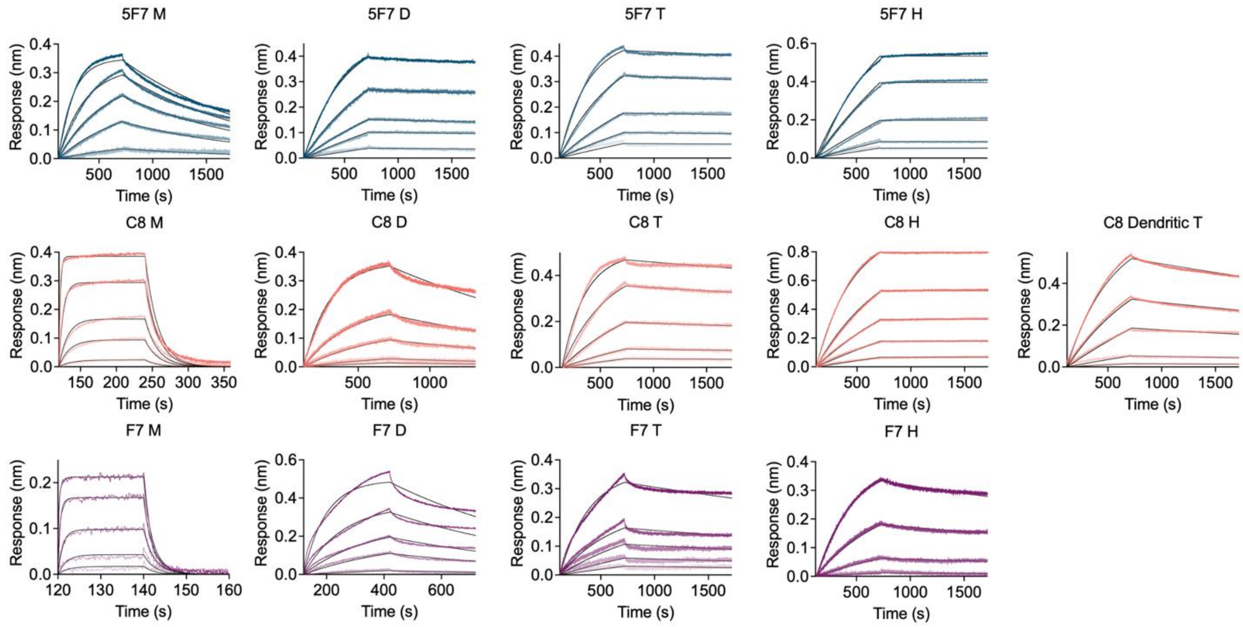

Figure S4. Biolayer interferometry (BLI) sensorgram traces of anti-HER2 M, D, T, H, and dendritic T binders to HER2 ECD.

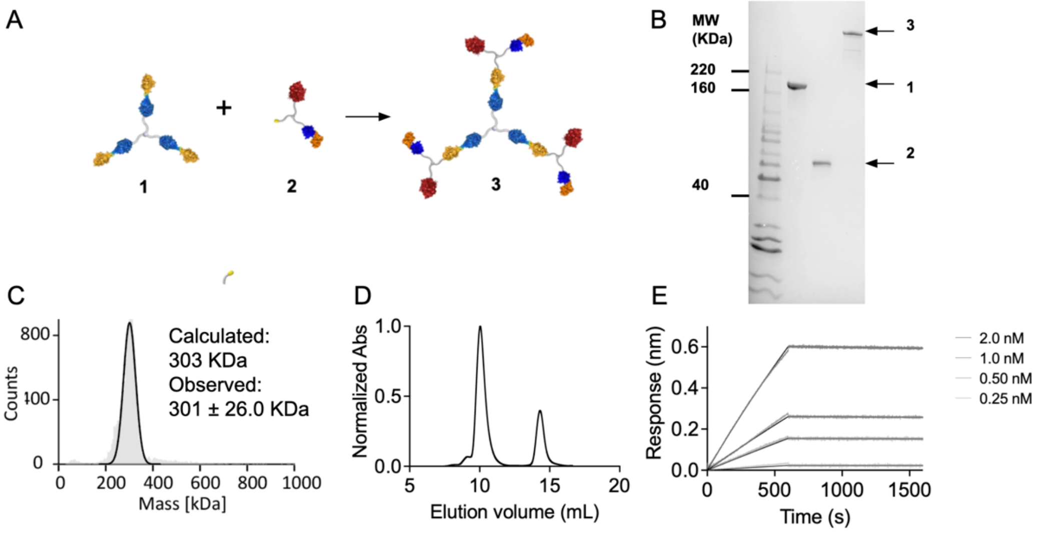

Figure S5. Synthesis and characterization of 7D12 dendritic T. (A) Synthesis scheme. (B) SDS-PAGE analysis. (C) Mass photometry analysis. (D) SEC purification trace. (E) BLI sensorgram trace.

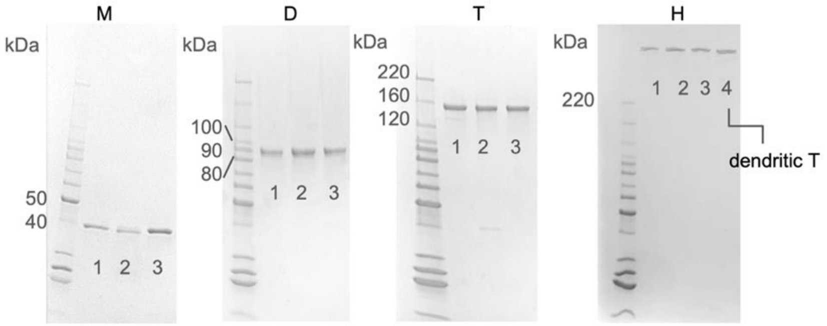

Figure S6. SDS-PAGE of anti-HER2 binders. Molecular weight markers are indicated next to the ladders.

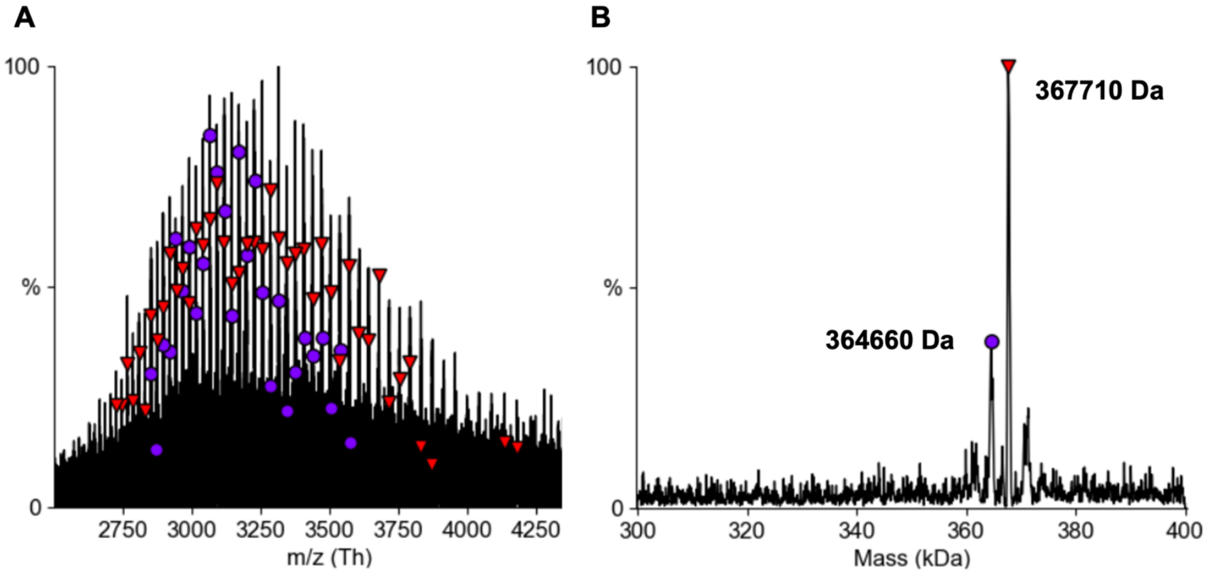

Figure S7. Native mass spectrometry spectra of 7D12 H. (A) Spectrum of the charge states. (B) Spectrum of the deconvoluted mass.

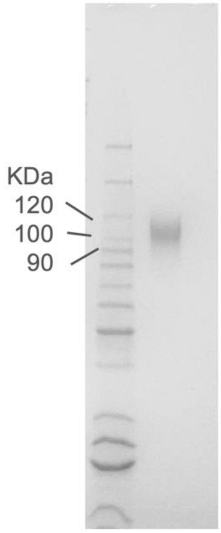

Figure S8. SDS-PAGE of EGFR ECD used in the mass photometry (MP) experiment. Due to abundant glycosylation of EGFR ECD and the presence of additional his- and AVI-tags, the observed molecular weight is higher than the theoretical value calculated from the amino acid sequence (71.9kDa, Met1-Ser645) and migrates as an approximately 100 kDa protein band.

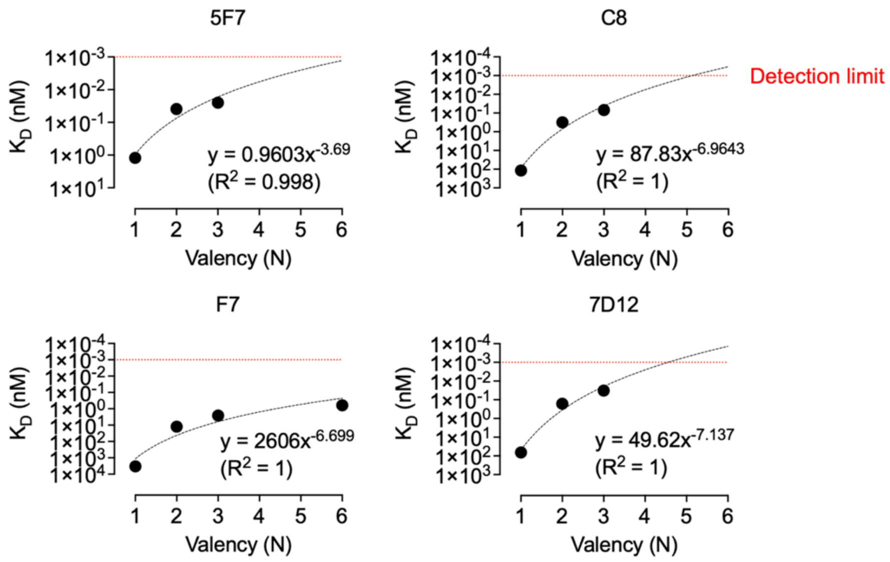

Figure S9. Binding affinity (K_D) values as a function of valency. K_D values for 5F7, C8, F7, and 7D12 were determined using biolayer interferometry (BLI) and plotted against binder valency. An inverse power law fit was applied to the detectable affinity data. K_D values for 5F7, C8, and 7D12 were not plotted as their affinities fell below the detection limit of the assay. The red dotted line denotes the BLI detection threshold (K_D < 0.001 nM).

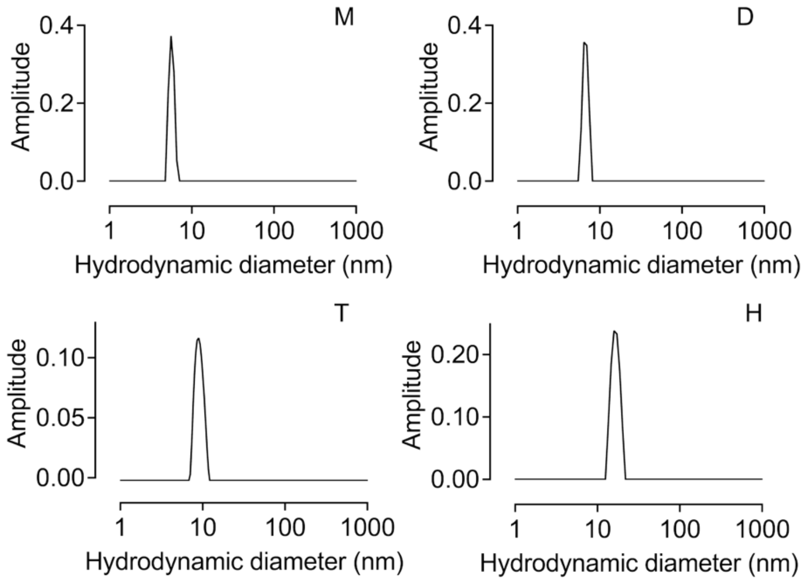

Figure S10. Dynamic light scattering (DLS) spectra of 7D12 M, D, T, and H.

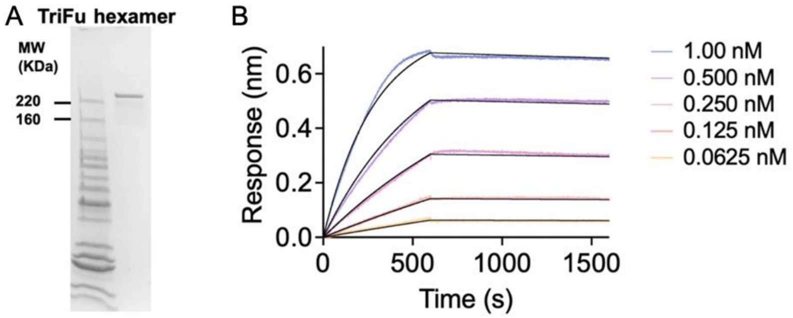

Figure S11. Characterization of the triple fusion hexamer (TriFu H). (A) SDS-PAGE analysis. ESI and MP measurements of molecular weight are summarized in Tables S2 and S3. (C) BLI sensorgram showing binding kinetics. The dissociation constant (K_D) was determined to be 8 pM, with an association rate constant (k_a) of 3.24×10^6 $M^{-1}s^{-1}$ and a dissociation rate constant (k_d) of 2.52×10^{-3} s^{-1}.

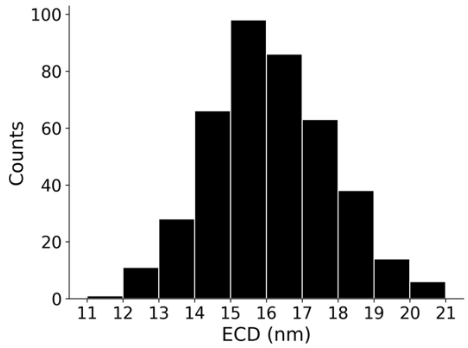

Figure S12. Particle size distributions for 7D12 H measured by transmission electron microscopy. Equivalent circular diameter (ECD) is used to represent the size of H binders with irregular shapes due to their flexibility.

Sequences of MegaMolecule Proteins

1. Cutinase – XTEN Linker – SnapTag – Histag (CS)
GLPTSNPAQELEARQLGRTTRDDLINGNSASCADVIFIYARGSTETGNLGTLGPSIASNLESAFGKDGVWI
QGVGGAYRATLGDNALPRGTSSAAIREMLGLFQQANTKCPDATLIAGGYSQGAALAAASIEDLDSAIRDKI
AGTVLFGYTKNLQNRGRIPNYPADRTKVFCNTGDLVCTGSLIVAAPHLAYGPDARGPAPEFLIEKVRAVR
GSASGSETPGTSESADKDCEMKRTTLDSPLGKLELSGCEQGLHEIIFLGKGTSAADAVEVPAPAAVLGGP
EPLMQATAWLNAYFHQPEAIEEFPVPALHHPVFQQESFTRQVLWKLLKVVKFGEVISYSHLAALAGNPAA
TAAVKTALSGNPVPILIPCHRVVQGDLDVGGYEGGLAVKEWLLAHEGHRLGKPGLGENLYFQGHHHHHH

2. 7D12 – XTEN Linker – Cutinase – Histag

MQVKLEESGGGSVQTGGSLRLTCAASGRTSRSYGMGWFRQAPGKEREFVSGISWRGDSTGYADSVKG
RFTISRDNAKNTVDLQMNSLKPEDTAIYYCAAAAGSAWYGTLYEYDYWGQGTQVTVSSSGSETPGTSES
AGLPTSNPAQELEARQLGRTTRDDLINGNSASCADVIFIYARGSTETGNLGTLGPSIASNLESAFGKDGV
WIQGVGGAYRATLGDNALPRGTSSAAIREMLGLFQQANTKCPDATLIAGGYSQGAALAAASIEDLDSAIR
DKIAGTVLFGYTKNLQNRGRIPNYPADRTKVFCNTGDLVCTGSLIVAAPHLAYGPDARGPAPEFLIEKVRA
VRGSAHHHHHH

3. Cutinase – XTEN Linker – 5F7 – Histag
GLPTSNPAQELEARQLGRTTRDDLINGNSASCADVIFIYARGSTETGNLGTLGPSIASNLESAFGKDGVWI
QGVGGAYRATLGDNALPRGTSSAAIREMLGLFQQANTKCPDATLIAGGYSQGAALAAASIEDLDSAIRDKI
AGTVLFGYTKNLQNRGRIPNYPADRTKVFCNTGDLVCTGSLIVAAPHLAYGPDARGPAPEFLIEKVRAVR
GSASGSETPGTSESAEVQLVESGGGLVQAGGSLRLSCAASGITFSINTMGWYRQAPGKQRELVALISSIG
DTYYADSVKGRFTISRDNAKNTVYLQMNSLKPEDTAVYYCKRFRTAAQGTDYWGQGTQVTVSSHHHHH
H

4. Cutinase – XTEN Linker – C8 – Histag
GLPTSNPAQELEARQLGRTTRDDLINGNSASCADVIFIYARGSTETGNLGTLGPSIASNLESAFGKDGVWI
QGVGGAYRATLGDNALPRGTSSAAIREMLGLFQQANTKCPDATLIAGGYSQGAALAAASIEDLDSAIRDKI
AGTVLFGYTKNLQNRGRIPNYPADRTKVFCNTGDLVCTGSLIVAAPHLAYGPDARGPAPEFLIEKVRAVR
GSASGSETPGTSESAVQLQASGGGFVQPGGSLRLSCAASGDSYNESSMGWFRQAPGKEREFVSAISA
RGNHPLYYADSVKGRFTISRDNSKNTVYLQMNSLRAEDTATYYCASMPMPKWKKYWGQGTQVTVSHH
HHHH

5. Cutinase – XTEN Linker – F7 – Histag
GLPTSNPAQELEARQLGRTTRDDLINGNSASCADVIFIYARGSTETGNLGTLGPSIASNLESAFGKDGVWI
QGVGGAYRATLGDNALPRGTSSAAIREMLGLFQQANTKCPDATLIAGGYSQGAALAAASIEDLDSAIRDKI
AGTVLFGYTKNLQNRGRIPNYPADRTKVFCNTGDLVCTGSLIVAAPHLAYGPDARGPAPEFLIEKVRAVR
GSASGSETPGTSESAVQLQASGGGFVQPGGSLRLSCAASGYSSAAEVMGWFRQAPGKEREFVSAISW
FHGETAYYADSVKGRFTISRDNSKNTVYLQMNSLRAEDTATYYCAENKPNEWGGQEMYWGQGTQVTV
SHHHHHH

7. CrabTag -Histag

PNFSGNWKIIRSENFEELLKVLGVNVMLRKIAVAAASKPAVEIKQEGDTFYIKTSTTVRTTEINFKVGEEFEE
QTVDGRPCKSLVKWESENKMVCEQKLLKGEGPKTSWTRELTNDGELILTMTADDVVCTRVYVREHHHH
HH

Additional Experimental Details

Materials. Sodium azide (NaN_3), sodium chloride, 2xYT media, LC-MS grade water, HisPur™ cobalt resin, Triton X-100, 10x PBS, bovine serum albumin (BSA) were purchased from Thermo Fisher Scientific. Kanamycin, isopropyl β-D-1-thiogalactopyranoside (IPTG), ammonium acetate, uranyl formate, apoferritin from equine spleen, thyroglobulin from bovine thyroid, and Amicon Ultra centrifugal filter units were purchased from Sigma Aldrich. Laemmli sample buffer and 4–15% precast protein gels were purchased from BIO-RAD. GBlocks gene fragments were purchased from Integrated DNA Technologies (IDT). The NEB Golden Gate Assembly enzyme mix (BsaI-HFv2), T4 DNA ligase buffer, and chemically competent cells (DH5α and Shuffle T7 Express) were purchased from New England Biolabs (NEB). Octet Streptavidin biosensors were purchased from Sartorius. Biotinylated EGFR Protein (ECD) and biotinylated HER2/ERBB2 Protein (ECD) were purchased from Sino Biological. TEM copper grids (CF300) were purchased from Electron Microscopy Sciences.
Cloning of fusion proteins. Gblocks gene fragments for the nanobody-Cutinase or Cutinase-SnapTag fusion proteins were obtained from IDT. Each Gblock included a BsaI restriction site and a start codon at the 5' end, followed by a hexa-histidine tag, a stop codon, and another BsaI restriction site at the 3' end. The Gblocks were digested with the NEB Golden Gate Assembly enzyme mix (BsaI-HFv2) in T4 DNA ligase buffer and subsequently ligated into a pET28b(+) plasmid following the manufacture's instruction. The resulting Golden Gate reaction mixture was transformed into the DH5α E. coli strain and plated on 2xYT agar supplemented with kanamycin. The plasmid sequences containing the target nanobody Cutinase fusion gene were confirmed and then transformed into the Shuffle T7 Express E. coli strain for subsequent protein expression.
Expression of Megamolecule Proteins. A 5 mL culture of 2×YT medium supplemented with 50 µg/mL kanamycin was inoculated with cell stocks expressing the fusion protein and incubated overnight (16–18 hours) at 30 °C with shaking at 250 rpm. This pre-culture was used to inoculate 500 mL of fresh 2×YT medium containing 50 µg/mL kanamycin. The larger culture was grown at 30 °C and 250 rpm until an optical density at 600 nm (OD_{600}) of 0.6–0.8 was reached (approximately 4.5 hours). Protein expression was induced by the addition of 0.25 mM IPTG, followed by incubation at 20 °C with shaking at 250 rpm for 16–18 hours. Cells were harvested by centrifugation at 7000 rpm for 10 minutes at 4 °C, and the resulting pellet was resuspended in PBS containing DNase I. Cell lysis was performed by sonication on ice for 10 seconds followed by a 20-second pause, repeated for a total of 3 minutes. The lysate was spun down at 4500 rpm for 10 minutes at 4 °C, and the supernatant was incubated with 3 mL of HisPur™ cobalt resin pre-equilibrated with PBS for 1 hour at 4 °C on a rotator. The protein-bound resin was washed three times with 10 mL of PBS and the protein of interest was eluted with 5 mL of 150 mM imidazole in PBS, repeated three times. The pooled eluates were concentrated to 5 mL using an Amicon centrifugal filter unit with a 10 kDa molecular weight cut-off, and further purified by size exclusion chromatography (HiLoad 16/60 Superdex 200) on an FPLC system in PBS containing 0.02% NaN_3. SEC fractions were analyzed by SDS-PAGE, and only those containing pure fusion protein were pooled for downstream applications.
Synthesis of Dimers (D). The nanobody–Cutinase fusion protein (20 µM, 2.2 eq) was reacted with a di-Cutinase homobifunctional EG7 linker (9.1 µM, 1 equivalent) in a total volume of 500 µL using PBS as the reaction buffer. The reaction mixture was purified by size exclusion chromatography (SEC) in PBS. SEC fractions were analyzed by SDS-PAGE, and those containing only the dimeric product were pooled and concentrated using ultrafiltration.
Synthesis of Trimers (T). The nanobody–Cutinase fusion protein (20 µM, 3.3 eq) was reacted with a tri-Cutinase homotrifunctional EG7 linker (6.1 µM, 1 eq) in a total reaction volume of 500 µL using PBS as the buffer. The reaction mixture was purified by size exclusion chromatography (SEC) in PBS. The collected SEC fractions were analyzed via SDS-PAGE, and those containing only the trimer product were pooled and concentrated by ultracentrifugation.
Synthesis of CS_3. The Cutinase-SnapTag fusion protein (20 µM, 4 eq) was reacted with a triCutinase homotrifunctional EG11 linker (5 µM, 1 eq) in a total reaction volume of 500 µL using PBS as the buffer. The reaction mixture was purified by size exclusion chromatography (SEC) in PBS. The collected SEC

fractions were analyzed via SDS-PAGE, and those containing only the trimer product were pooled and concentrated by ultracentrifugation.

Synthesis of Hexamers (H). The nanobody-Cutinase (Nb) fusion protein (20 μM, 2.2 eq) was reacted with a Cutinase-Cutinase-SnapTag hetero-trifunctional EG7 linker (9.1 μM, 1 eq) in a total volume of 5 mL using PBS as the buffer. The reaction mixture was purified by size exclusion chromatography (SEC) in PBS, and fractions containing the dimer product (Nb-Nb-s), consisting of two nanobody domains linked by a linker with a free SnapTag-inhibitor, were pooled and concentrated by ultracentrifugation. The purified Nb-Nb-s (20 μM, 6 eq) was then reacted with CS_3 (3.3 μM, 1 eq) in a total volume of 2 mL in PBS supplemented with 1 mM EDTA, which was included to prevent precipitation caused by interactions between His-tags and trace metal ions The mixture was purified by SEC in PBS with 1mM EDTA and the collected fractions were analyzed by SDS-PAGE. Fractions containing only the hexamer product were pooled and concentrated by ultracentrifugation.

Synthesis of Dendritic Trimers (Dendritic T). The nanobody–Cutinase (Nb) fusion protein (7D12 or C8) (20 μM, 1.1 eq) and CrabTag (R) (27.3 μM, 1.5 eq) were reacted with a Cutinase–CrabTag–SnapTag hetero-trifunctional linker (18 μM, 1 eq) in a total volume of 5 mL using PBS supplemented with 0.2% Tergitol as the reaction buffer. The mixture was purified by size-exclusion chromatography (SEC) in PBS, and fractions containing the desired dimeric product (Nb–R–s), comprising one nanobody domain and one CrabTag domain linked via the trifunctional linker bearing a free SnapTag inhibitor, were pooled and concentrated by ultracentrifugation. The purified Nb–R–s (9 μM, 6 eq) was subsequently reacted with CS_3 (1.5 μM, 1 eq) in a total reaction volume of 500 μL in PBS supplemented with 1 mM EDTA to prevent precipitation. The final reaction mixture was purified by SEC in PBS containing 1 mM EDTA, and the resulting fractions were analyzed by SDS-PAGE. Fractions containing only the dendritic T product were pooled and concentrated by ultracentrifugation.

Synthesis of 7D12 TriFu H. The 7D12 triple fusion protein (20 μM, 6 eq) was reacted with a triCutinase homotrifunctional EG7 linker (3.3 μM, 1 eq) in a total reaction volume of 500 μL using PBS as the buffer. The reaction was purified by size-exclusion chromatography (SEC) in PBS, and the collected fractions were analyzed by SDS-PAGE. Fractions containing only the TriFu H product were pooled and concentrated by ultracentrifugation.

Sodium Dodecyl Sulfate-Polyacrylamide Gel Electrophoresis (SDS-PAGE). Protein samples were mixed with Laemmli buffer and heated at 95°C for 5 minutes. The samples were then loaded into 15- or 30-well, 4-15% Tris-glycine precast gels and ran at 150 V for 55 minutes. The gels were stained with Coomassie blue for visualization.

Electrospray Ionization Mass Spectrometry (ESI). ESI spectra were acquired using an Agilent 6545 LC-QTOF mass spectrometer equipped with an Agilent Series 1200 HPLC binary pump and autoinjector. Protein stock solutions were diluted in nuclease-free water to a final concentration of 1 μM. A 4 μL aliquot of each sample was injected into a 1 cm C18 guard column and eluted using a linear gradient from 0% to 100% acetonitrile containing 0.1% formic acid over 7.5 minutes, at a flow rate of 0.4 mL/min. Data were analyzed using Agilent MassHunter BioConfirm software.

Dynamic light scattering (DLS). The 7D12 monomer, dimer, trimer, and G1 hexamer were diluted to a final concentration of 24 μM, 12 μM, 8 μM, 2.5 μM, respectively. Each sample (6 μL) was placed in a rUNt cuvette and measured on pUNk using the default settings (10 acquisitions of 10 seconds each) at 22 ℃ to obtain the Z-average diameter.

Mass Photometry (MP). The measurements were performed using a Refeyn TwoMP mass photometer. A solution of 7D12 H (160 nM) was incubated with a sixfold molar excess of EGFR extracellular domain (EGFR ECD) at room temperature for 60 minutes. For each measurement, 19.4 μL of filtered PBS was placed onto a silicone gasket mounted on a microscope slide to focus the instrument. Subsequently, 0.6 μL of the 7D12 H–EGFR ECD mixture was added to the same well, mixed thoroughly, and a movie was recorded for 60 seconds. Samples were independently prepared and measured in quadruplicate. Contrast values were converted to molecular weights using a calibration curve generated with Thyroglobulin (670 kDa), Apoferritin (480 kDa), IgG (150 kDa), and BSA (66 kDa), with calibrations performed every two hours to ensure accuracy. The populations corresponding to EGFR ECD, 7D12 H, and 7D12 H–EGFR complexes were quantified by fitting the MP distributions with Gaussian models using DiscoverMP software.

Negative-stain Transmission Electron Microscopy. A 4 μL aliquot of 7D12 H protein solution (25 nM) was applied to a carbon-supported, 300-mesh copper grid that had been negatively glow-discharged. After a 30-second incubation, the grid was blotted and washed three times with 100 μL of Milli-Q water,

with blotting after the final wash. The grid was then stained with 4 μL of 0.75% (w/v) uranyl formate, blotted, and stained again with an additional 12 μL of uranyl formate for 60 seconds before final blotting. The grid was air-dried for 15 minutes prior to imaging. Transmission electron microscopy was performed using a 300 kV JEOL ARM300F Grand ARM TEM at the NUANCE Center at Northwestern University. For particle size distribution analysis, 412 particles were manually selected, and their areas were measured using ImageJ. The equivalent circular diameter (ECD) for each particle was then calculated from the measured area.

Native Mass Spectrometry. A 150 mM ammonium acetate solution was prepared using LC-MS grade Optima™ water and filtered through 0.2 μm sterile disposable filters. The 7D12 H solution was concentrated to 1.2 mg/mL (3.3 μM) in PBS, with a final volume of 100 μL. It was then dialyzed for 1 hour against 1 L of 150 mM ammonium acetate solution using a Slide-A-Lyzer Dialysis unit with a 10 kDa molecular weight cut-off for direct infusion of electrospray ionization. The sample was sprayed via a Nanospray Flex source and a medium-sized borosilicate-coated emitter into a Q Exactive Ultra High Mass Range (UHMR) mass spectrometer (Thermo Fisher Scientific). A spray voltage of +2.4 kV, an in-source collision-induced dissociation value of 15 V, and a source temperature of 320 °C were utilized to desolvate ions. The resolution was 280,000 at 200 m/z. Spectra (1,500-11,000 m/z) were accumulated for 10 min. Data was integrated and deconvoluted using UniDec (https://unidec.chem.ox.ac.uk/).

Biolayer interferometry (BLI). Binding analyses were performed using the Octet K2 Bio-Layer Interferometer. Biotinylated EGFR extracellular domain (EGFR ECD; 3 μg/mL) or biotinylated HER2 ECD (1 μg/mL) was immobilized onto Streptavidin (SA) biosensors for 240 seconds, achieving a final signal of 1–1.2 nm. Association and dissociation measurements were conducted at 30 °C in PBS supplemented with 0.04% Triton X-100. For high-affinity constructs (all 5F7 variants, as well as T, H, and dendritic T constructs), an association phase of 600 seconds and a dissociation phase of 1000 seconds were used. The total experimental duration was kept under 2 hours to minimize sample evaporation and maintain data accuracy. Baseline subtraction was applied to correct for signal drift throughout the measurements. Binding affinities (K_D), association rate constants (k_a), and dissociation rate constants (k_d) were calculated using the Octet data analysis software. Curve fitting was performed using a 1:1 binding model based on pseudo-first-order kinetics. All kinetic and affinity parameters were derived from a global fit with an R^2 value of at least 0.95 to ensure robustness and reliability. Detailed conditions for each binder, including association/dissociation times and concentrations, are summarized in Table S6.

Synthesis of Megamolecule Linkers. Cutinase-SnapTag-CrabTag heterotrifunctional linker (tri-EG(7,7,7)-(pNPP, CP, R)) was synthesized as previously described.[23] CrabTag inhibitor (R), SnapTag inhibitor (CP), and Cutinase inhibitor (pNPP) used in the linker synthesis were prepared according to the previously reported procedures (Figure S10). [20, 23, 64]

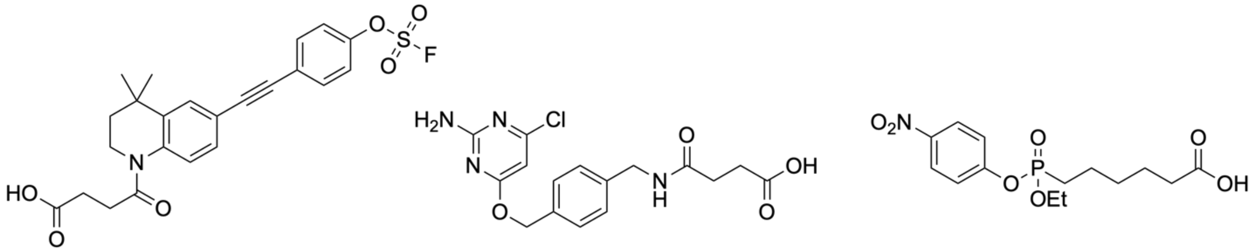

Figure S10. Structure of *p*-nitrophenyl phosphonate (left, pNPP), chloro-pyrimidine (middle, CP), and synthetic retinoid (right, R).

DiCutinase linker (pNPP – EG7 – pNPP). The linker was synthesized according to previously published method.[21] Amino-EG7-amine was used as the linker spacer and only *p*-nitrophenyl phosphonate was used to produce the homo-bifunctional linker. ^{1}H NMR (500 MHz, MeOD) δ 8.46 – 8.20 (m, 4H), 7.54 – 7.39 (m, 4H), 4.33 – 4.15 (m, 4H), 3.71 – 3.58 (m, 22H), 3.55 (t, *J* = 5.5 Hz, 4H), 3.37 (t, *J* = 5.5 Hz, 4H), 2.23 (t, *J* = 7.4 Hz, 4H), 2.14 – 2.00 (m, 4H), 1.78 – 1.62 (m, 9H), 1.50 (qd, *J* = 7.3, 4.1 Hz, 4H), 1.35 (t, *J* = 7.1 Hz, 6H). MALDI-MS m/z 1045.21 ($[M + Na]^+$, calcd for $C_{44}H_{72}N_4O_{19}P_2$ 1022.43).

Cutinase-Cutinase-SnapTag linker (Tri-EG(7, 7, 7)-(pNPP, pNPP, CP)). The linker was synthesized according to previously published method and azido-EG7-amine was used during the solid-phase synthesis step.[27] ^{1}H NMR (500 MHz, MeOD) δ 8.36 (s, 3H), 8.24 – 8.15 (m, 4H), 7.38 – 7.31 (m, 4H), 7.31 – 7.25 (m, 2H), 7.23 – 7.16 (m, 2H), 6.02 (s, 1H), 5.23 (s, 2H), 4.25 (s, 2H), 4.18 – 4.03 (m, 4H), 3.60

– 3.44 (m, 85H), 3.43 – 3.39 (m, 6H), 3.25 – 3.22 (m, 5H), 2.42 (t, *J* = 3.6 Hz, 4H), 2.10 (t, *J* = 7.4 Hz, 4H), 2.01 – 1.90 (m, 4H), 1.65 – 1.57 (m, 4H), 1.56 – 1.47 (m, 4H), 1.41 – 1.32 (m, 4H), 1.22 (t, *J* = 7.1 Hz, 6H). MALDI-MS m/z 2284.17 ($[M + Na]^+$, calcd for $C_{101}H_{159}ClN_{12}O_{39}P_2$ 2261.00).

TriCutinase linker (Tri-EG(7, 7, 7)-(pNPP, pNPP, pNPP)) The linker was synthesized according to previously published method. [27] Azido-EG7-amine and *p*-nitrophenyl phosphonate was used during the solid-phase synthesis step. ^1H NMR (500 MHz, MeOD) δ 8.47 (s, 3H), 8.35 – 8.27 (m, 6H), 7.46 (dd, *J* = 9.3, 1.2 Hz, 6H), 4.33 – 4.14 (m, 6H), 3.78 – 3.45 (m, 96H), 2.22 (t, *J* = 7.5 Hz, 6H), 2.14 – 2.00 (m, 6H), 1.79 – 1.60 (m, 12H), 1.53 – 1.44 (m, 6H), 1.34 (t, *J* = 7.1 Hz, 9H). MALDI-MS m/z 2243.30 ($[M + H]^+$, calcd for $C_{99}H_{162}N_9O_{42}P_3$ 2242.00).

TriCutinase linker (Tri-EG(11, 11, 11)-(pNPP, pNPP, pNPP)) The linker was synthesized according to previously published method. [27] Azido-EG11-amine and *p*-nitrophenyl phosphonate was used during the solid-phase synthesis step. ^1H NMR (500 MHz, $CDCl_3$) δ 8.51 (s, 3H), 8.22 (d, *J* = 8.6 Hz, 6H), 7.37 (d, *J* = 8.6 Hz, 6H), 4.26 – 4.10 (m, 6H), 3.61 (m, 142H), 3.42 (q, *J* = 5.3 Hz, 6H), 2.17 (t, *J* = 7.4 Hz, 6H), 1.93 (m, 6H), 1.76 – 1.63 (m, 12H), 1.44 (m, 6H), 1.31 (t, J = 7.1 Hz, 8H). MALDI-MS m/z 2771.26 ([M + H]+, calcd for $C_{123}H_{210}N_9O_{54}P_3$ 2770.32).

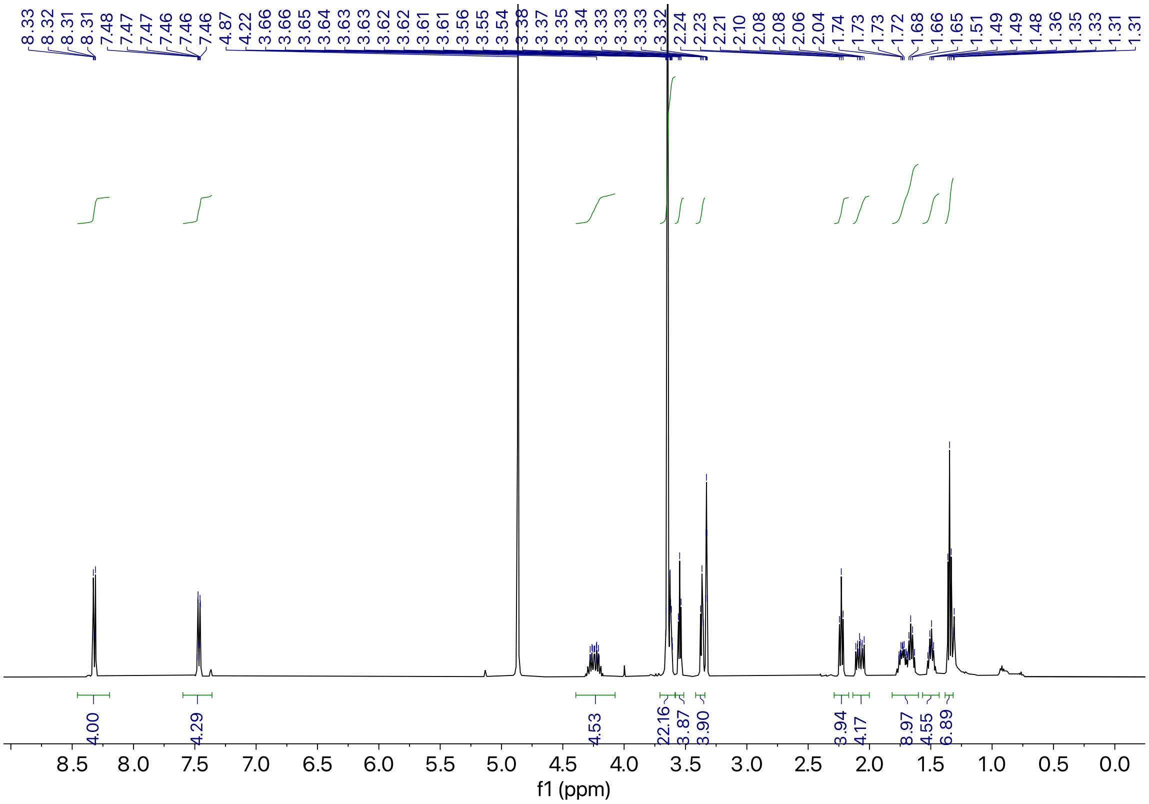

Figure S11. ^1H NMR spectrum of pNPP – EG7 – pNPP in MeOD.

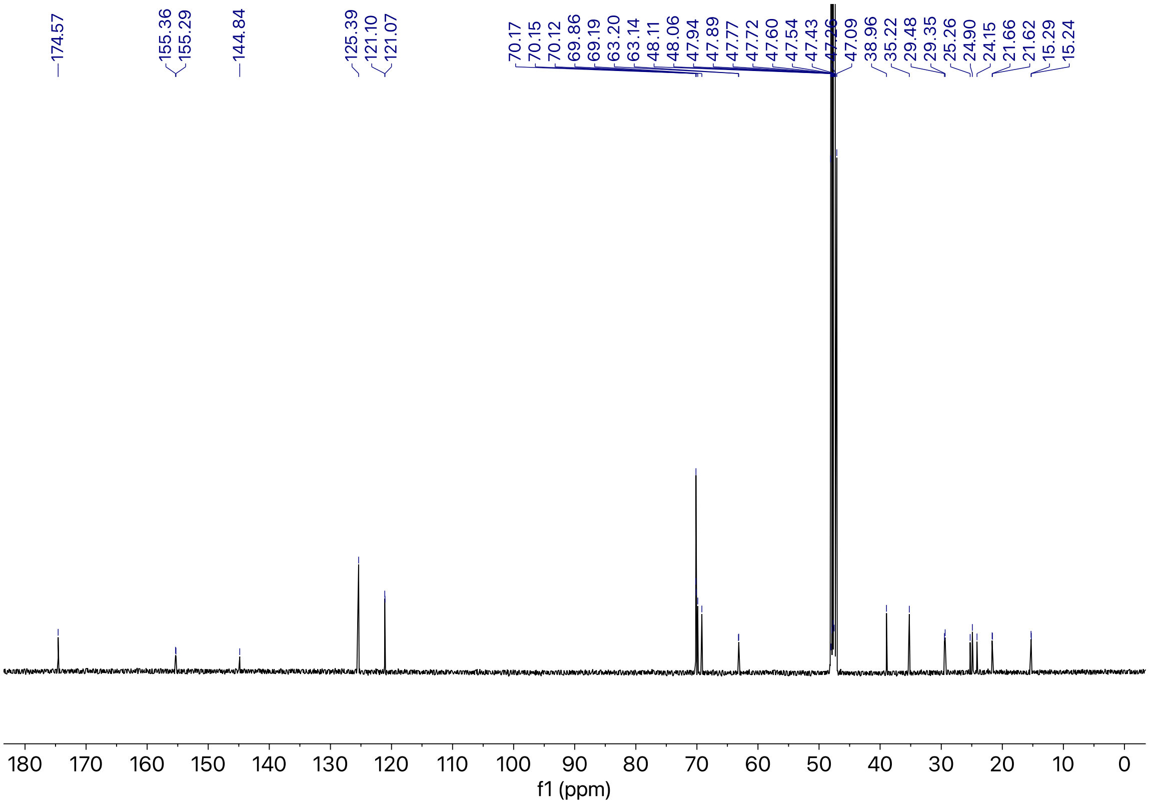

Figure S12. ^{13}C NMR spectrum of pNPP – EG7 – pNPP in MeOD.

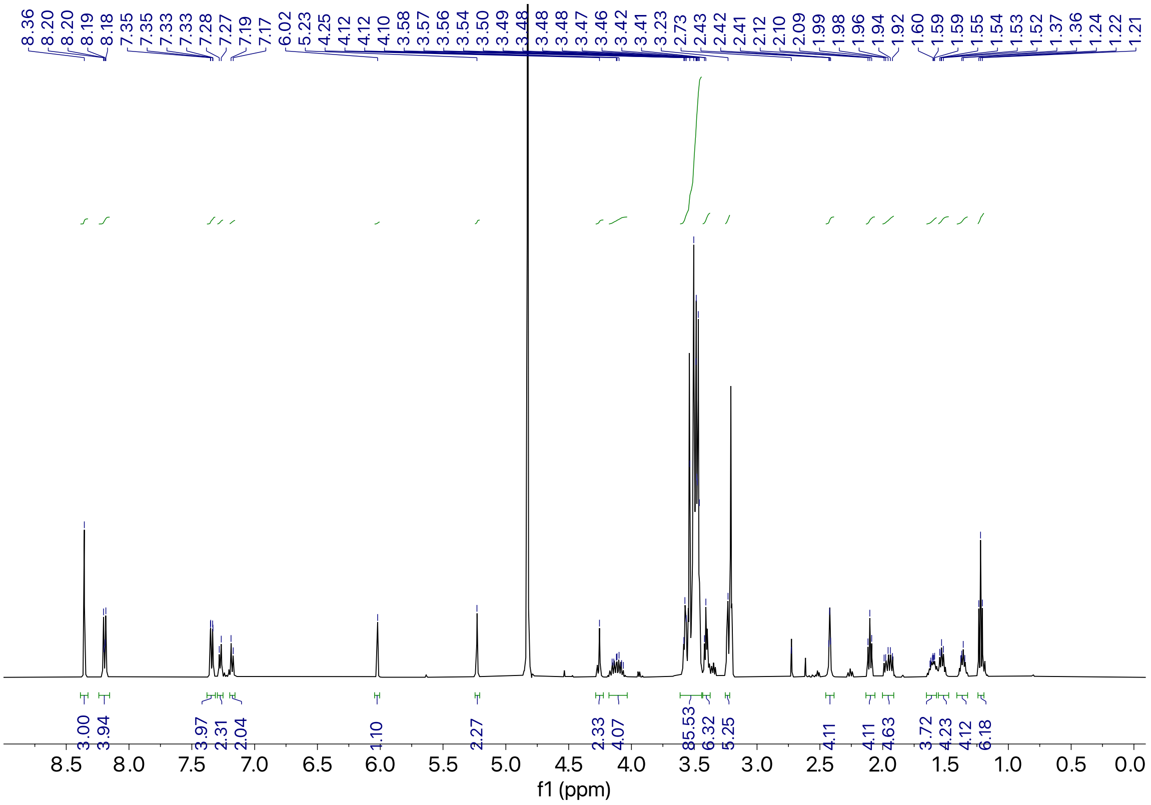

Figure S13. ^{1}H NMR spectrum of Tri-EG(7,7,7)-(pNPP, pNPP, CP) in MeOD.

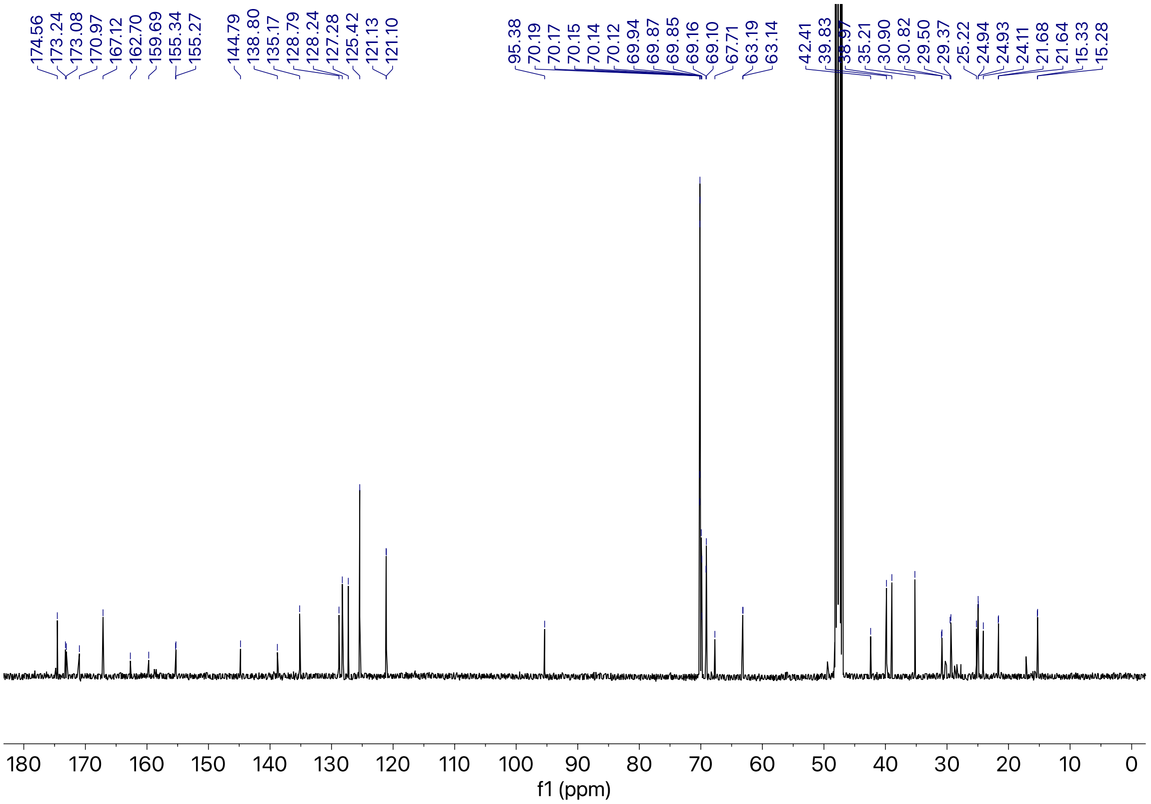

Figure S14. ^{13}C NMR spectrum of Tri-EG(7,7,7)-(pNPP, pNPP, CP) in MeOD.

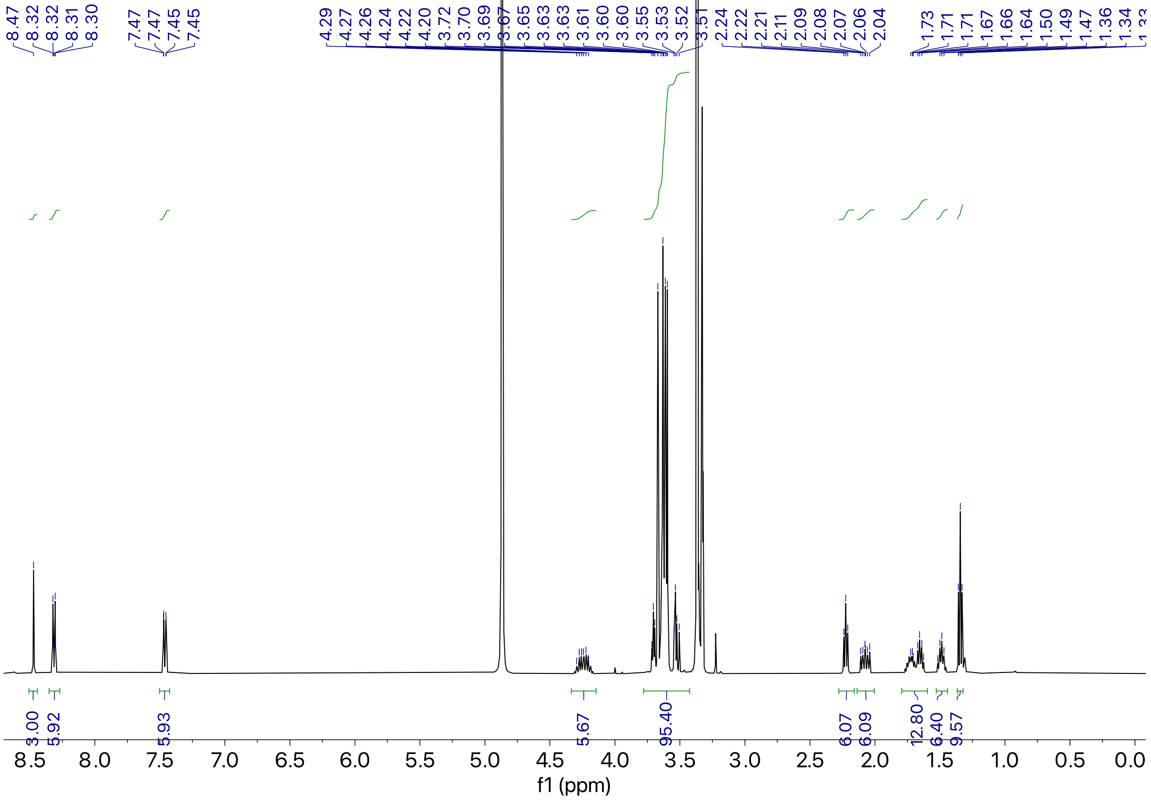

Figure S15. ^{1}H NMR spectrum of Tri-EG(7,7,7)-(pNPP, pNPP, pNPP) in MeOD.

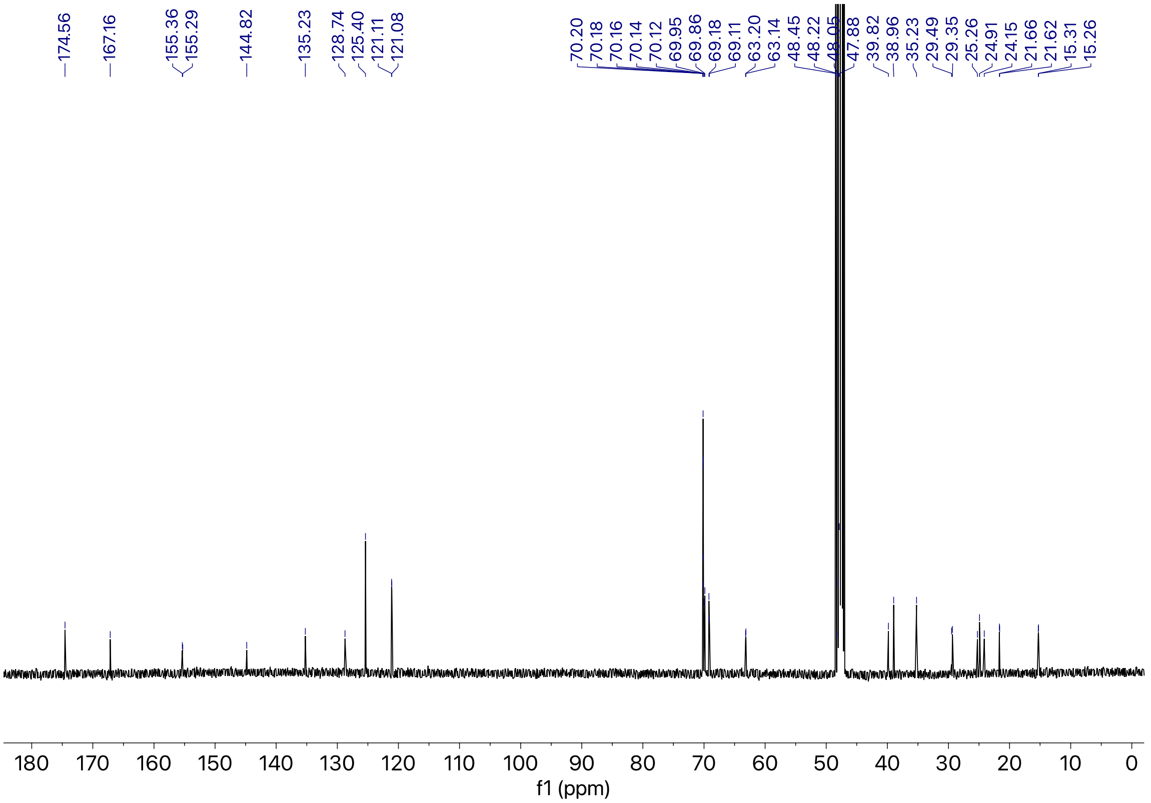

Figure S16. ^{13}C NMR spectrum of Tri-EG(7,7,7)-(pNPP, pNPP, pNPP) in MeOD.

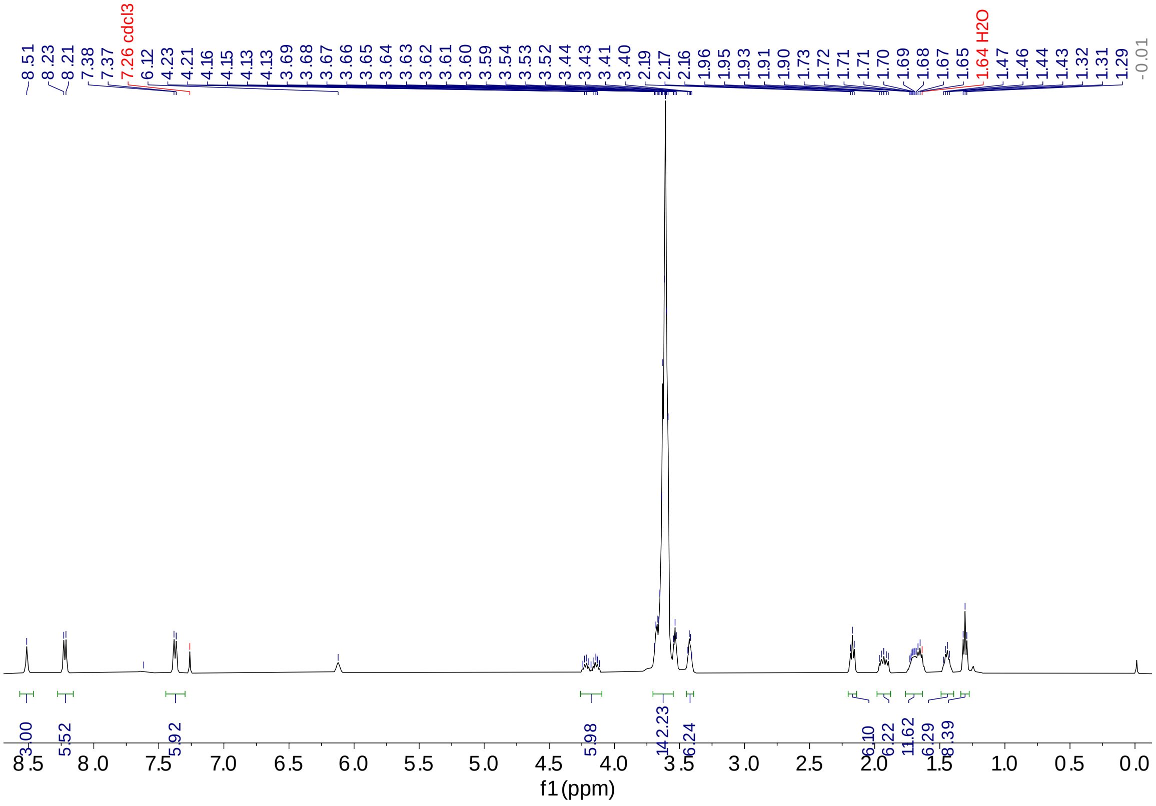

Figure S17. ^{1}H NMR spectrum of Tri-EG(11,11,11)-(pNPP, pNPP, pNPP) in $CDCl_3$.
